# Roles of MAPKs, Including Those Activated by BDNF/TrkB, and Their Contribution in Neurodegenerative Diseases

**DOI:** 10.3390/ijms27020984

**Published:** 2026-01-19

**Authors:** Tadahiro Numakawa, Ryutaro Kajihara

**Affiliations:** 1 Department of Cell Modulation, Institute of Molecular Embryology and Genetics, Kumamoto University, Kumamoto 860-0811, Japan; 2 Department of Hematology and Immunology, Faculty of Life Science, Kumamoto University, Kumamoto 862-0976, Japan

**Keywords:** ERK1/2, MAPK, BDNF, Alzheimer’s disease, neurodegeneration, Parkinson’s disease

## Abstract

Brain-derived growth factor, BDNF, has critical roles in a wide variety of neuronal aspects, including cell survival, differentiation, and synaptic function after their maturation. TrkB, a high-affinity receptor for BDNF, is a major contributor in these neuronal aspects, and its functions are exerted via stimulating intracellular signaling pathways including the mitogen-activated protein kinase (MAPK) pathways. As a family of MAPKs, the functions of ERK1/2, p38MAPK, and JNKs have been extensively studied using in vivo and in vitro neuronal systems. ERK 1/2, a major serine-threonine kinase and belonging to the MAPK family, also works as a downstream molecule after activation of the BDNF/TrkB system. Interestingly, growing evidence has demonstrated that ERK1/2 signaling exerts a positive or negative influence on neurons in both healthy and pathological conditions in the central nervous system (CNS). Indeed, activation of ERK 1/2 stimulated by the BDNF/TrkB system is involved in the regulation of synaptic plasticity. On the other hand, overactivation of ERK1/2 signaling under pathological conditions is closely related to neurodegeneration. Furthermore, cell stress activates p38MAPKs and JNK signaling, contributing to the progression of neurodegeneration. In this review, we show how MAPK pathway signaling affects neuronal fate, including cell survival or cell death, in the CNS. Moreover, we discuss the involvement of overactivation of MAPK signaling in the neurodegeneration observed in Alzheimer’s disease (AD), Parkinson’s disease (PD), and Huntington’s disease (HD).

## 1. Introduction

It has been well-established that decreased levels of BDNF in the brain are involved in the pathogenesis of neurodegenerative diseases including AD, PD, and HD because BDNF and its related molecules are essential for various neural events including cell proliferation, survival, synaptic function, and neurogenesis by activating the intracellular signaling such as MAPK signaling pathway [1]. In addition to BDNF, nerve growth factor (NGF), which was found as a neurotrophin first, also promotes neuronal cell survival in the peripheral nervous system (PNS) and CNS neurons [1]. Furthermore, neurotrophin-3 (NT3) is also a member of neurotrophin, and NT4/5 has also been identified. As a specific high-affinity receptor, it has been established that the TrkA receptor is a specific one for NGF, and TrkC is for NT3, although TrkB is for both NT4/5 and BDNF. Trk receptors are a family of tyrosine kinase receptors, and trigger intracellular signaling pathways such as PLCγ, PI3K/Akt, and MAPK, affecting cell fate decisions in the PNS and CNS. In addition to Trk receptors, the low-affinity p75NTR is also involved in neurotrophin actions because the receptor binds to all neurotrophins [1,2].

MAPK/ERKs signaling contributes to various cellular programs such as cell proliferation, cell differentiation, and cell survival maintenance. Although, as the family of MAPKs, ERK1/2, p38MAPK, and JNKs have been extensively studied, ERK1/2 was first identified [3]. ERK1/2 are one of Serine/Threonine (Ser/Thr) protein kinases and are stimulated by a variety of extracellular signals including the BDNF/TrkB system or oxidative stress. ERK1/2, one of the most investigated kinases, are involved in synaptic regulation, learning, and memory functions [4,5]. For example, in cultured neurons, BDNF induced the upregulation of synaptic proteins including synapsin I, and glutamate receptor subunits (NR2A, NR2B, GluR1), by activating ERK1/2, suggesting positive effects of ERK1/2 on the synapse formation [6]. In contrast, 17beta-estradiol protected cultured neurons against oxidative stress by reducing activation of ERK1/2 [7]. Therefore, ERK1/2 has both positive and negative influences on neurons.

ERK1/2 were reported to be involved in the regulation of neural gene expression, neurotransmission, and changes of synapses, suggesting that the ERK1/2 pathway has a role in the learning and memory function [4]. Interestingly, an abnormal activation (hyperactivation) of ERK1/2 can contribute to disease progression (see [8]). Generally, it has been recognized that parallel activation of ERK1 and ERK2 is induced by cellular stimulants of the ERK1/2 signaling pathway [9]. A lot of effort to find differences among ERK1 and ERK2 revealed that the functions of the two isoforms are very similar, although a few studies suggested that the two isoforms are not entirely redundant [9]. Actually, almost all studies on stress-related MAPK pathways seem to evaluate ERK1 and ERK2 equivalently. In the CNS, ERK1/2 are one of the contributors to brain development, and regulate synaptic functions, maintaining healthy memory formation, while the kinases can cause an enhancement of cell death induction and neuroinflammation (see [8]). Oxidative stress, neuroinflammation, apoptosis, and ferroptosis are suggested to affect the progression of traumatic brain injury, and hyperactivated ERK1/2 potentiates oxidative stress, inflammation, and neuronal cell death [10]. Importantly, evidence has demonstrated that a variety of stressors in the CNS neurons, such as oxidative stress and neuroinflammation, contribute to the AD pathophysiology. Remarkably, it has been well known that subfamilies of MAPKs (p38MAPKs, JNKs, and ERK1/2) are associated with an infinite number of physiological events in non-neuronal cells [11]. Since ERK1/2 signaling is important for cell division, it is possible that inhibitors for ERK1/2 have been considered as anticancer drugs [11]. In addition, cell stress and inflammatory cytokines elicit activation of p38MAPKs, suggesting a critical contribution of the p38MAPK signaling in the regulation of autoimmunity. Furthermore, the inhibition of JNK signaling is effective in ameliorating rheumatoid arthritis [11].

**Figure 1:**
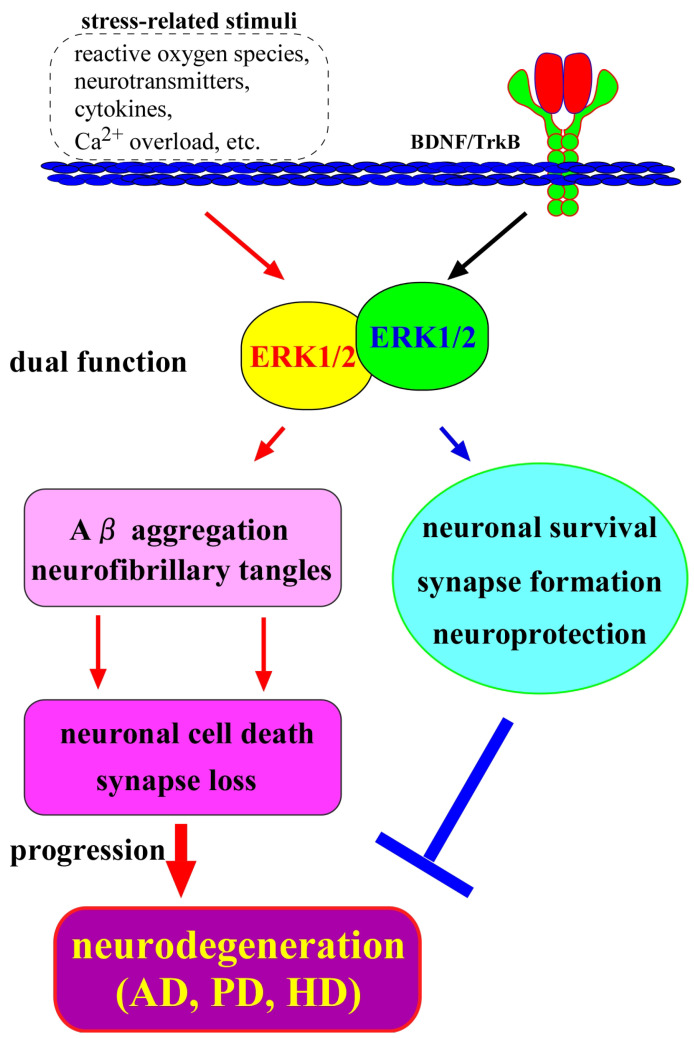
Roles of ERK1/2 and BDNF/TrkB in the CNS neurons. Extracellular regulated kinase 1/2 (ERK 1/2), which are well-studied serine-threonine kinases, are stimulated by a variety of extracellular stimuli, and their hyperactivation is known to be involved in the enhancement of pathological conditions including Aβ plaques production, resulting in neurodegeneration. On the other hand, an activation of the ERK1/2 pathway occurs as one of the downstream signaling pathways of the BDNF/TrkB and has a role in the maintenance of CNS neurons. Hyperactivation of ERK1/2 signaling caused by various extracellular stimuli (not by the BDNF/TrkB system) has been suggested to induce synapse loss and/or neuronal cell death, as confirmed in neurodegenerative diseases such as AD, PD, and HD.

Because evidence suggests a hyperphosphorylation state of various MAPKs, including ERK1/2, in a variety of pathogenesis conditions reflects the neurodegeneration, a detailed understanding of the MAPK signaling dynamics in the CNS neurons is required. In this review, we show recent studies on the positive and negative influence of MAPKs (for example, stimulated form by the BDNF/TrkB system) and their overactivation under the pathological conditions, which is suggested to be closely related to neurodegeneration (see Figure 1). Furthermore, we discuss the status of MAPKs when applying drug candidates in the model for AD, PD, and HD.

## 2. Relationship Among ERK Signaling and BDNF/TrkB System in Neurons

As mentioned above, it has been suggested that downregulation of the neurotrophins and/or their Trks have roles in the AD pathogenesis. Interestingly, using cell lines, cultured cortical neurons, and animals, Fernandez et al. (2023) examined the effects of ACD856, which is a novel allosteric modulator for Trks [12]. It was revealed that ACD856 enhanced neurite outgrowth induced by NGF in PC12 cells. In primary cortical neurons, ACD856 upregulated levels of pERK1/2, and an Aβ-caused decrease in SNAP25-positive neurites was restored by ACD856 treatment [12]. Further, it was shown that the administration of ACD856 upregulated levels of BDNF protein in the brains of aged animals (21-month-old mice) [12]. Using rats, Kim et al. (2014) showed beneficial effects of monoclonal antibody 29D7, an agonist for TrkB, against perinatal hypoxic ischemia (H-I) [13]. After an intracerebroventricular (icv) administration of 29D7, marked activation of ERK1/2 and Akt in neurons was confirmed. Importantly, when rats received 29D7 administration and were subjected to H-I with unilateral carotid artery ligation and 8% oxygen hypoxia exposure, an activation of caspase-3 (as a hallmark of apoptosis) caused by H-I injury was significantly suppressed by 29D7 [13]. In addition to the BDNF/TrkB system, to improve the low bioavailability and its BBB impermeability when using the NGF/TrkA system, the effects of ENT-A013, which is one of the NGF mimetics and a selective activator of TrkA, were examined and confirmed its neuroprotective and anti-Aβ function as a potential therapeutic candidate against AD [14]. After injury and/or disease, the transplantation of neural stem cells (NSCs) is expected to induce neural repair; thus, Chen et al. (2017) investigated whether NSCs, which were genetically modified to overexpress BDNF (BDNF/NSCs), are effective in synaptogenesis in the rat model of traumatic brain injury (TBI) [15]. As expected, the transplantation of BDNF/NSCs significantly upregulated the TrkB gene and increased the levels of pTrkB in the lesions caused by TBI. In addition to increased PSD95, levels of Ras and pERK1/2 were also elevated in the BDNF/NSCs transplantation groups compared with those in the naive NSCs ones [15]. These studies demonstrate that ERK1/2 signaling stimulated by the BDNF/TrkB system has a positive effect on the maintenance of CNS neurons (see Figure 1).

## 3. Natural Compounds and ERK Signaling in AD Models

Growing evidence has demonstrated the beneficial effects of natural compounds mediating ERK1/2 signaling in AD models. Choi et al. (2025) recently reported that paeoniflorin (PF) exerted an inhibitory effect against amyloidogenesis and neuroinflammation [16]. The amyloidogenesis and neuroinflammation observed in BV-2 microglial cells treated with lipopolysaccharide (LPS) were improved by the application of PF. Furthermore, using an amnesic mouse model, they found that deficits in the memory function of animals were improved by PF [16]. In the hippocampus and cerebral cortex of the animals, although increased pp38MAPK, pJNK, and pERK1/2 caused by LPS were reduced by PF, upregulated pCREB and BDNF, and decreased APP were induced after PF application [16]. Phylloporia ribis (PRG), one of the traditional Chinese medicines, has been used to improve weakness and memory loss in aged persons. Indeed, a recent study showed that PRG exerted neuroprotective effects using an in vitro AD model produced by Aβ25-35 exposure [17]. PC12 cells exposed to Aβ25-35 exhibited increased mitochondrial stress and subsequent apoptosis, which were all reversed by PRG, with upregulation of pERK, pCREB, and BDNF proteins, suggesting that PRG restored an inhibition of the ERK pathway [17]. Furthermore, Cornuside, an extract obtained from Corni Fructus, improved the decreased learning and memory in D-galactose (D-Gal)-treated mice [18]. Cornuside upregulated BDNF, and downregulated the RAGE, Iba1(for microglial), and GFAP (for astrocyte) in the hippocampus and cortex of the D-Gal-treated animals. Remarkably, in addition to pERK1/2, the phosphorylation levels of JNK and p38 MAPK were significantly increased by D-Gal administration, although only pERK1/2 was dramatically decreased by Cornuside treatment, suggesting a contribution of overactivation of ERK1/2 in neuron damage caused by D-Gal [18]. Using Aβ-treated rats, Kuedo et al. (2022) showed beneficial effects of ethanolic extract of white shrimp (*Litopenaeus vannamei*) shells (EESS) against memory impairment [19]. Interestingly, treatment with EESS alone failed to improve; however, the EESS-loaded liposome significantly improved memory ability in Aβ-treated animals [19]. The EESS-loaded liposome increased protein levels of BDNF, TrkB, GAP-43, and PSD-95 as well as pERK1/2 in the cortex and hippocampus of Aβ-treated animals [19]. Cycloastragenol (CAG), which is a triterpenoid saponin and an ingredient in Astragalus membranaceus (Fisch.) Bunge, is known as one of the Chinese medicinal herbs [20]. An increasing body of evidence has demonstrated that CAG has various pharmacological effects, including anti-oxidative properties, anti-inflammatory, and especially telomerase activation [20]. Ikram et al. (2021) examined the effects of CAG against Aβ-induced AD-like phenotypes in animals [21]. It was revealed that stereotaxic injection of Aβ caused oxidative stress and decreased expression of BDNF and pTrkB. As expected, decreased expression of NeuN, a neuron marker, in the cortex and hippocampus of Aβ-injected mice was observed. When CAG was given in Aβ-injected mice, BDNF, pTrkB, and NeuN were significantly reversed [21]. Interestingly, the upregulations of pJNK, pp38MAPK, and pERK1/2 induced by Aβ injection were significantly reduced by CAG treatment [21]. Using cultured hippocampal neurons, Munni et al. (2023) examined the effects of Garlic (*Allium sativum* L.) on neurite outgrowth and synapse development [22]. They compared the effects of the ethanol extracts of unprocessed (white garlic extract, WGE) and processed (black garlic extract, BGE) garlic on hippocampal synaptogenesis. Neurite outgrowth was enhanced by both WGE and BGE without cytotoxicity, although the more robust effects of WGE were found [22]. Significant upregulation of both NR2A and NR2B was induced by WGE and BGE, but the increased level of NR2A by BGE was higher. The analysis with gas chromatography and mass spectrometry revealed that both extracts contained linalool, which has significant neurite outgrowth effects in the cultured neurons. By using network pharmacology, they demonstrated that the effects of linalool were exerted via regulating signaling pathways, including GSK3β and ERK1/2 [22]. Interestingly, an enhancement of the BDNF/TrkB system with flavonoids (small molecules activating TrkB signaling) has been intensively studied (See [2]). Beneficial effects of a variety of flavonoids against the progression of AD have been demonstrated. Tang et al. (2025) recently reported the influence of isoliquiritigenin, which is a flavonoid obtained from the root of liquorice, in various aspects of AD models [23]. Treatment with isoliquiritigenin significantly restored the spatial memory capacity of mice receiving streptozotocin injection. Levels of pTau and reactive oxygen species in the cortex and hippocampus of the AD model mice caused by streptozotocin were improved by isoliquiritigenin. Furthermore, isoliquiritigenin restored synaptic impairment and loss of neurons and decreased the levels of mTOR and ERK activity [23]. A recent study also showed that glycosaminoglycans have protective effects against neurotoxicity caused by fluoride exposure [24]. Chondroitin sulfate (CS, one of the glycosaminoglycans) improved the deficits in learning and memory of rats that received a fluoride exposure [24]. Decreased hippocampal synaptophysin (a synaptic marker) caused by fluoride was reversed by CS. Although upregulation of metalloproteinase-9 (MMP-9), total ERK1/2, and pERK1/2 in vivo and in vitro (using SH-SY5Y cells) after fluoride exposure occurred, CS significantly reversed these fluoride-dependent negative effects, suggesting that CS rescued impaired synaptic functions by fluorosis exposure through repressing the ERK1/2-MMP-9 pathway [24].

These studies suggest that the upregulation of the BDNF/TrkB system after treatment with a variety of natural compounds is considered a strong method to inhibit AD pathogenesis. However, the activation status of ERK1/2 does not always correspond to that in the BDNF/TrkB system. Among the compounds, the actions of flavonoids in the disease models seem to be similar to those of the BDNF/TrkB system (See [2]).

## 4. Chemicals and ERK Signaling in AD Models

Joodi et al. (2025) reported that cabergoline (CAB, a dopamine receptor agonist) improved deficits in spatial and recognition memory using ovariectomized rats injected with D-Gal [25]. The concentrations of β-secretase, Aβ42, and pTau in the hippocampus of the model rats were decreased by CAB administration, although pCREB and BDNF were increased by CAB. Importantly, upregulation of glutamate transporter-1 protein, which promoted uptake of glutamate contributing to Ca2+ overload and consequently reduced the phosphorylated forms of p38MAPK and ERK1/2, was observed after CAB treatment [25]. A recent study has shown that (2R,6R)-hydroxynorketamine (HNK, a ketamine metabolite) reversed decreased protein synthesis and synaptic plasticity in the hippocampus of AD mouse models [26]. Hippocampal LTP and memory deficits caused by Aβ oligomers infusion were restored by HNK [26]. In addition, ERK1/2, mTOR, and p70S6 kinase 1 (S6K1)/ribosomal protein S6 signaling pathways were activated by treatment with HNK. HNK also rescued synaptic and memory defects and corrected transcriptional alterations in the hippocampus of the APP/PS1 AD mice [26]. Because chemotherapy may contribute to the long-term cognitive deficits in breast cancer survivors, Salas-Ramirez et al. (2015) studied possible mechanisms underlying cognitive impairments when chemotherapeutic agents were used to treat the cancer. Ovariectomized (OVX) and intact female rats were treated with saline or a combination of doxorubicin and cyclophosphamide. Significantly impaired working and spatial memory by chemotherapy were observed. Interestingly, no difference in the impaired memory among OVX and intact animals was confirmed [27]. On the other hand, an increased pERK1/2 and pAkt, as well as PSD95 expression in OVX female rodents after the chemotherapy was induced, although OVX females displayed higher levels of BDNF compared with those in intact ones, independent of chemotherapy [27]. Senescence-accelerated mouse-prone 8 (SAMP8) mice exhibit significant age-related deficits in learning and memory function in compliance with early onset of senescence [28,29]. It has been demonstrated that SAMP8 mice are useful to investigate the mechanisms of senescence acceleration, the onset of AD, and other cognitive disorders. Recently, because the majority of AD cases are sporadic due to unknown genetic causes, SAMP8 mice, which display Aβ deposits, hyperphosphorylated Tau, inflammation, and neuronal cell death, can be useful for clarifying features of sporadic AD [28]. Lian et al. (2021) showed the therapeutic effects of DL0410, a novel acetylcholinesterase inhibitor, using SAMP8 mice. It was revealed that DL0410 administration significantly improved the cognitive deficits in SAMP8 mice [30]. DL0410 increased synaptic proteins, including PSD95 and synaptophysin, in the mouse brain. Interestingly, DL0410 administration upregulated BDNF and TrkB, and the neurotrophic effects were mediated via the ERK1/2 and PI3K/Akt/GSK3β pathways [30]. Zhang et al. (2020) examined the effects of DL0410 against lipopolysaccharides (LPS)-induced neuroinflammation and hydrogen peroxide-induced oxidative stress, as both neuroinflammation and oxidative stress are involved in AD [31]. They found that DL0410 reduced both inflammatory responses and ROS production in BV2 cells treated with LPS [31]. DL0410 restored downregulated pCREB, pTrkB, and pERK1/2 and expression of BDNF [31].

When using AD models, the status of pERK1/2 often differs from that in the BDNF/TrkB system (see Figure 1). The hyperactivation of ERK1/2 signaling is caused by reactive oxygen species, excess neurotransmitters, or cytokines, etc. (Figure 1) [32,33]. It has been speculated that ERK1/2 hyperactivation is often correlated with synaptic dysfunction and neuronal apoptosis. Further interventions concerning the relationship among upregulation of BDNF (or status of pTrkB) and ERK1/2 signaling are required.

## 5. Inhibitors of ERK Signaling and AD Models

Recently, Hassan et al. (2025) examined the effects of limettin (5,7-dimethoxycoumarin) on AD-like pathology using mouse models [34]. In their system, sporadic AD (SAD) animal models were established using an injection of streptozotocin (STZ, a compound derived from Streptomyces achromogenes), which induced activation of microglial cells, causing neuroinflammation and oxidative [35,36].

The treatment with limettin and PD98059 (a specific inhibitor for MEK) reversed deficits in cognitive and memory performance of the SAD animals [34]. Furthermore, limettin and PD98059 decreased persistent activation of pERK1/2 in the hippocampus of the SAD mice. Importantly, the enhancement of pCREB (Ser133) and BDNF expressions was observed in SAD mice receiving PD98059 and limettin [34]. In addition to the ERK1/2 pathway, p38 MAPK signaling is also involved in the pathophysiology of AD since Aβ peptide and/or tauopathies stimulate activation of the p38 MAPK [37]. For example, a significant improvement in associative and spatial memory deficit observed in APP/PS1 Tg AD mice was achieved by treatment with MW01-18-150SRM, a selective inhibitor of p38αMAPK [38]. Using APP/PS1 knock-in mice, it was also revealed that spatial memory deficit in the AD animals was ameliorated by the p38αMAPK inhibitor [38].

## 6. Neurotoxicity of Aβ and ERK Signaling

Previously, Morroni et al. (2016) showed the early memory deficits caused by soluble beta-amyloid oligomers (AβO). They also reported that cognitive decline in mice receiving intracerebroventricular injection of AβO was associated with elevated caspase-9 activation and oxidative stress, and decreased immunoreactivity of synaptophysin in the hippocampus of the animals [39]. In their system, AβO injection caused an increased ERK1/2 activity [39]. It was reported that expression levels of serine/arginine repetitive matrix 2 (SRRM2) and polyglutamine binding protein 1 (PQBP1, the splicing regulatory protein relating to an intellectual disability) were slightly lower in the postmortem cerebral cortex in AD patients, and that ERK1/2 had a role in phosphorylation of SRRM2 at Ser1068 [40]. Interestingly, before Aβ aggregation in the AD model mice, the subcellular localization shift (from the nucleus to the cytoplasm) of SRRM2 occurred when SRRM2 was phosphorylated [40]. It was revealed that the SRRM2 localization shift decreased PQBP1, impairing synapses. suggesting ERK signaling and splicing-related proteins play a role in the early-phase pathologies of AD [40]. As mentioned above, it has been demonstrated that dysregulation of ERK1/2 is involved in cognitive impairments and neuropathogenesis of AD. A recent report showed that E-twenty-six (ETS)-like protein 1 (ELK1), a member of transcription factors, which is activated by ERK1/2, has a role in AD pathogenesis. Yi et al. (2025) reported that ELK1 knockdown or inhibiting its activation via an interfering peptide decreased amyloidogenic processing of APP, consequently reducing the generation of Aβ in APP23/PS45 double-transgenic AD animals [41]. Interestingly, the suppression of ELK1 expression or activation improved synaptic and memory impairments observed in the AD animal, suggesting a possible contribution of ERK1/2 to AD pathogenesis through regulating ELK1 function [41]. Using the AlCl3-induced AD rat models, Salama et al. (2025) examined the effects of betanin (BET)-loaded liposomal nanocarriers (LPN, to enhance the brain penetration and bioavailability, on AD-like behaviors [42]. In addition to less cortical and hippocampal degeneration, an improvement in spatial and learning memory was achieved by BET-LPN treatment compared with oral BET treatment. Furthermore, BET-LPN treatment reduced the levels of ERK1/2 and MEK1/2 with more effectiveness compared with traditional oral BET [42]. Wu et al. (2025) investigated the influence of long-term cervical lymphadenectomy (cLE) in AD-like tauopathy [43]. They found that male mice that received cLE displayed significant impairment in cognitive function and anxiety-depressive behaviors. Significant impairment in brain waste (including Aβ and pTau drainage), which activated ERK1/2, leading to reduced autophagy, was caused by CLE [43]. To investigate the possible impact of prenatal stress (PS) on brain maturation and cognitive function in offspring, Trojan et al. (2023) used male C57BL/6 J (WT) and the knock-in (KI) APPNL-F/NL-F mice carrying Swedish and Beyreuther/Iberian mutations, and pregnant animals were exposed to daily restraint stress (E12.5–E18.5) [44]. They found an increased level of ApoE and Aβ42/Aβ40 in the frontal cortex and the hippocampus of male offspring of the KI animals [44]. Furthermore, a changed insulin signaling, an interference in ERK1/2- and mTOR-signaling, and pro-inflammatory (IL-6, IL-23, and TNF-α) status were observed in the KI mice [44]. Remarkably, a recent study using single-cell (scRNA-seq) and single-nucleus (snRNA-seq) RNA sequencing demonstrated cellular and molecular alterations when mice received distal middle cerebral artery occlusion (dMCAO) [45]. In this experiment, animals were assigned to control, sham, 3-day-dMCAO, and 14-day-dMCAO. Cell populations, including neurons (both glutamatergic and GABAergic ones), astrocytes, oligodendrocytes, microglia, endothelial cells, fibroblast-like cells, and pericytes were identified by snRNA-seq [45]. Importantly, neuronal death, autophagy, and cAMP biosynthesis pathways were shown by Gene Ontology analysis. Increased levels of syngap1, Rock1, and Ikbkb in the glutamatergic populations suggested a contribution to vulnerability to ischemic injury. Dissociation of SynGAP1 from PSD-95 after ischemia may increase pERK1/2 levels, while ischemic preconditioning reduces the dissociation and inhibits ERK1/2 overactivation [45].

Under pathological conditions, it has been demonstrated that hyperactivation of ERK1/2 (or sustained activation of ERK1/2) is recognized as one of the contributors to potentiate the progression of AD pathophysiology. Using a pathological system in which ERK1/2 is a contributor, further investigation concerning drug candidates that increase both BDNF- and ERK1/2-signaling may be interesting.

## 7. JNK Signaling in AD Models

As mentioned above, it is well known that AD displays Aβ deposition and tau hyperphosphorylation as pathology-specific hallmarks, and neuroinflammation and oxidative stress are involved in the pathogenesis of the disease. As it has been demonstrated that natural products are therapeutic candidates for AD treatment, recent advances in the beneficial effects of various natural compounds (including flavonoids and phenolic compounds) are very interesting [46]. Especially, evidence has shown that these natural compounds regulate ERK, p38MAPK, and JNK pathways, and thereby suppress oxidative stress and neuroinflammation, leading to attenuating synaptic dysfunction and neuronal cell loss (see [46]). Concerning JNKs, there are three isoforms, JNK1, JNK2, and JNK3, which are a subfamily of MAPKs, function as one of the key mediators in the cellular responses to inflammation and signals of cell death [47]. Both JNK1 and JNK2 are expressed across tissues broadly. JNK1 and JNK2 have critical roles in immune signaling, cell proliferation, and death. Expression of JNK3 is restricted to the heart, testis, and brain, suggesting its contribution to the pathophysiology of neurodegeneration observed in AD. Indeed, evidence suggests that JNK3 is involved in the pathogenesis of the neurodegenerative disorder via enhancing neuroinflammation, oxidative stress, and neuronal cell death [47].

Recently, the neuroprotective effects of ambroxol in mice treated with scopolamine to cause oxidative stress-related neurodegeneration were reported [48]. After 2 weeks of scopolamine and/or ambroxol, levels of ROS and lipid peroxidation (LPO) were increased by scopolamine administration in the cortex and hippocampus; however, treatment with ambroxol significantly reduced these ROS and LPO levels [48]. Furthermore, increased pJNK and decreased pAkt and glycogen synthase kinase-3 beta (GSK-3β) induced by scopolamine were improved by ambroxol. The mice’s behavioral performance tests (Morris water maze and Y-maze paradigms) revealed ameliorating effects of ambroxol treatment against the impairments in spatial learning and memory caused by scopolamine [48]. Using 5xFAD mice, the protein expression of BDNF and JNK after the administration of a water-extracted roasted Astragali radix (RA, one of the traditional oriental medicines) has been shown [49]. Behavioral change evaluated using the open field test (OFT), Morris water maze (MWM), Y-maze, elevated plus maze (EPM), active avoidance (AA) tasks 8 weeks after the initiation of RA administration (5 months old mice) revealed an improvement in the spatial memory, working memory, fear avoidance memory, and lowered anxiety in the AD mice [49]. Interestingly, significant upregulation of BDNF and pCREB, and downregulation of pJNK in the hippocampus were induced by RA treatment [49].

In vitro and in vivo analysis revealed an involvement of JNK signaling in the protective effects of C66, an analogue of curcumin, against the pathology of AD [50]. Significant cytotoxicity in PC12 cells and primary neurons was caused by Aβ, with an increased JNK activation. Importantly, C66, or SP600125, a specific JNK inhibitor, attenuated the Aβ-induced cytotoxicity with an inhibition of the JNK pathway [50]. Furthermore, chronic C66 administration improved a cognitive decline (NOR and MWM test) in APP/PS1 mice. In the hippocampus of AD animals, reduced levels of Aβ plaques (sAPPβ, Aβ42, and Aβ40) and attenuated JNK signaling were achieved by C66 treatment [50]. Muraleva et al. (2025) examined age-related changes in hippocampal JNK signaling of Wistar rats and senescence-accelerated OXYS rats, which display accelerated symptoms of AD-like pathology [51]. They investigated changes in the JNK signaling using males of OXYS rats and control Wistar rats 20 days, and 4.5, 5, and 18 months of age, respectively. It was revealed that, with age, the activity of the JNK signaling increased in the hippocampus of rats of both strains. Furthermore, when IQ-1S, an inhibitor of JNK3, was administered to 4.5 to 6-month-old Wistar and OXYS rats, a significant decrease in the phosphorylation levels of JNK3, c-Jun, APP, and Tau in the hippocampus was confirmed, suggesting that the JNK signaling is a potential target for delaying neurodegenerative progression [51].

The neurotrophic system includes BDNF/TrkB and p75NTR/sortilin (high-affinity receptor for proBDNF, a precursor protein of BDNF), and plays critical roles in neuronal survival and apoptosis, affecting neurodegenerative outcomes (see [52]). It has been well known that the BDNF/TrkB system stimulates survival-promoting pathways such as PI3K/Akt and MAPK/ERK. On the other hand, evidence suggests that activation of the p75NTR/sortilin system by proBDNF results in neuronal apoptosis via stimulating JNK signaling (see [52]). Further studies concerning the relationship between hyperactivation of JNKs and the neurotrophic system (BDNF/TrkB and p75NTR/sortilin) using each pathological experiment system may be interesting.

## 8. p38MAPK-Signaling in AD Models

Critical role of p38MAPKs in both physiological and disease conditions, such as AD models, has been examined (see [53]). Although the four p38MAPK isoforms, including p38α, p38β, p38γ, and p38δ, are expressed in the whole mouse brain and cerebellum, isoforms of p38α and p38β localize in the murine hippocampus and neocortex (see [53]). Especially, the p38α isoform has been studied most extensively, and its functions in CNS neurons have been demonstrated [53].

Using PC12 cells damaged by exposure to Aβ25-35, Xu et al. (2025) examined the therapeutic effects of Huangqi San (HQS), a classical traditional Chinese medicine formula, on AD [54]. Interestingly, it was revealed that HQS significantly attenuated neurotoxicity caused by Aβ25-35 treatment. The Aβ25–35 administration decreased levels of pPI3K, pAKT, and pERK1/2, although increased levels of pJNK and pp38MAPK were induced by the Aβ administration. Importantly, the increased pERK1/2 and decreased pJNK and pp38MAPK were reversed by HQS [54], suggesting different roles of these MAPK families in the PC12 cells damaged by Aβ25-35. Rafi et al. (2025) reported neuroprotective effects of quinic acid (1) and its amide derivatives (2–4) against neurotoxicity caused by phytohaemagglutinin (PHA) [55]. It was revealed that treatment of the SH-SY5Y cells with the respective compounds 1 and its derivatives (2–4) for 48 h increased the cell viability. Interestingly, 1 and its derivatives (2–4) decreased the elevated levels of inflammatory molecules (IL-1β and TNF-α) induced by PHA. The compounds also reduced increased levels of Aβ, pTau, and pp38MAPK [55]. Recently, a beneficial effect of ethanol extract of leaves of Perilla frutescens var. acuta Kudo, a member of the Lamiaceae family, against neuroinflammation was shown [56]. Production of NO and pro-inflammatory mediators (TNF-α, IL-6, NF-κB, iNOS, and COX-2) in BV2 microglial cells exposed to LPS was significantly reduced by the standardized 60% ethanol extract of Perilla leaves (PE) [56]. An inhibition of the LPS-induced activation of JNK and p38MAPK was also reduced by PE. Furthermore, using AD animals made by intracerebroventricular injection of Aβ, an improvement in cognitive function (Morris water maze, novel object recognition, passive avoidance, and Y-maze tests) by PE was confirmed [56]. Because the Yangming–Kaixin–Yizhi formula (YKY), which has been clinically applied for the treatment of memory loss-related disorders, Lei et al. (2025) examined effects of YKY against AD phenotype in 3xTg-AD mice [57]. As expected, YKY significantly improved learning and memory function (Morris water maze) in the AD mice. Furthermore, increased hippocampal Iba-1 and GFAP-positive cells in the AD animals were reduced by YKY treatment. Reduced activation of PI3K/Akt and increased activation of p38MAPK in the hippocampus of the AD mice were reversed by YKY [57]. Lee et al. (2025) showed decreased short-term spatial/recognition memory (Y-maze and novel object recognition tests), and increased levels of pp38MAPK when overexpressing dual-specificity tyrosine phosphorylation-regulated kinase 1A (DYRK1A) in the hippocampus of wild mice [58]. Conversely, when the knockdown of DYRK1A in Aβ-overexpressing 5xFAD mice was performed, improved short-term spatial/recognition memory and increased levels of pCaMKIIα and pCREB were observed [58].

Recently, inhibitors for p38MAPK have been targeted to repress neuroinflammation in AD. Samir et al. (2025) reported the preventive effects of selective p38αMAPK inhibitors on neuroinflammation [59]. They designed and synthesized specific inhibitors of p38α, which were derivatives of benzimidazole-aminopyrimidine-hybrids and showed weaker activity on p38β, p38γ, and p38δ. Using AD rat models, which were produced by daily intraperitoneal injection of AlCl_3_, they found that treatment with p38α inhibitors significantly reduced brain levels of pro-inflammatory cytokines (NF-κB, TNF-α, and IL-1β) and exerted notable histopathological improvement in cortical and hippocampal regions in the AD models [59]. Interestingly, it is possible that cholesterol homeostasis is involved in tau phosphorylation. When the knockdown of 24-dehydrocholesterol reductase (DHCR24), which has a pivotal role in cholesterol biosynthesis, was carried out in SH-SY5Y cells, in addition to reduced cellular cholesterol levels, significantly increased tau phosphorylations (Thr181, Ser199, and Ser202/Thr205) were induced [60]. Furthermore, the DHCR24-silenced cells displayed increased pp38MAPK and pJNK. Importantly, SB203580, a p38MAPK inhibitor, or SP600125, a JNK inhibitor, attenuated the tau phosphorylation, suggesting that p38MAPK and JNK signaling pathways contribute to the tau pathology mediated by the DHCR24-controlled cholesterol homeostasis [60]. Hu et al. (2025) also identify nilotinib as a small-molecule disruptor for p38/MK2 (MAPK-activated protein kinase2) protein-protein interaction, leading to suppression of cytokine induction in microglia [61].

These studies, using a variety of in vivo and in vitro models, suggest that the upregulation of the p38MAPK system contributes to AD pathogenesis. However, an interaction of p38MAPK signaling with the status of the ERK1/2 system may be interesting to clarify the molecular mechanism under the progression of AD pathogenesis.

## 9. Role of MAPK Signaling in Parkinson’s Disease

PD affects more than 10 million individuals worldwide and is clinically defined by motor symptoms such as bradykinesia, rigidity, and resting tremor, as well as non-motor manifestations including cognitive impairment and mood disturbances [62]. The pathological hallmarks of PD include degeneration of nigrostriatal dopaminergic neurons and intracellular accumulation of α-synuclein aggregates, known as Lewy bodies. Despite substantial advances in symptomatic therapy, including dopamine replacement strategies, no current treatment effectively halts or reverses neurodegeneration [62].

As mentioned above, neurotrophic factors have emerged as critical regulators of neuronal integrity in both the developing and adult brain. Among them, BDNF has received particular attention due to its high expression in dopaminergic circuits and its ability to promote neuronal survival through activation of TrkB [63]. Downstream of TrkB, the MAPK/ERK pathway transduces extracellular cues into transcriptional programs that support neuronal plasticity, differentiation, and resistance to oxidative or proteotoxic stress. Understanding how ERK signaling mediates BDNF’s neuroprotective effects may reveal novel therapeutic strategies for PD.

BDNF supports the survival of dopaminergic neurons in the substantia nigra and enhances the functional activity of striatal medium spiny neurons, the primary target neurons receiving input from the substantia nigra. It has also been shown that BDNF facilitates both the synthesis and uptake of dopamine [63].

Multiple lines of evidence indicate that BDNF signaling is disrupted in PD. For example, postmortem studies reveal reduced BDNF and TrkB expression in PD brains, particularly in the substantia nigra and striatum [64,65,66]. Another study identified a nigrostriatal phenotype in aged haploinsufficient TrkB mutant mice (TrkB^+/−^) that mirrors a preclinical stage of PD [67]. This phenotype is characterized by a reduction of tyrosine hydroxylase (TH)-positive neurons in the substantia nigra pars compacta (SNpc), accompanied by a loss of striatal TH-positive fibers and extensive α-synuclein accumulation within dopaminergic cell bodies [67]. The downregulation of this trophic axis may exacerbate neuronal vulnerability to mitochondrial dysfunction and oxidative damage, both of which are central to PD pathogenesis [2]. Thus, the impairment of BDNF signaling represents not only a consequence but also a contributing factor to dopaminergic degeneration.

Experimental models using α-synuclein fibrils or overexpression demonstrate attenuated ERK activation. A recent study reported that aberrant phosphorylation of α-synuclein at Ser129 (pS129) inhibited activation of the BDNF/ERK signaling pathway in PD model rats [68]. Pharmacological activation of ERK1/2 alleviated the pathological effects of pS129, restored BDNF expression in the medial prefrontal cortex (mPFC), and reduced pS129 levels in both the substantia nigra and mPFC [68]. Another study demonstrated that transfection with constitutively active MEK-1 induced ERK phosphorylation in cells overexpressing α-synuclein, which consequently enhanced cell viability, thereby reinforcing the neuroprotective function of ERK1/2 signaling [69].

However, the role of ERK1/2 in PD is complex and context-dependent: while transient activation exerts protective effects, sustained or aberrant activation, often induced by neurotoxins like MPP^+^ or 6-hydroxydopamine (6-OHDA), can lead to neuronal stress and apoptosis [70]. For example, activated ERK1/2 has been observed in Lewy bodies, the pathological hallmarks of PD, composed of aggregated α-synuclein within degenerated neurons [71]. Another experimental PD model also showed that exposure to 6-OHDA induced sustained ERK1/2 activation, whereas treatment with PD98059, a MEK inhibitor, mitigated cell death [72]. Using a human iPSC-based PD model, a recent study demonstrated, through transcriptomic profiling and subsequent biochemical validation, that the ERK1/2 pathway is activated in cortical neurons derived from PD patients [73]. They further showed that inhibition of this signaling cascade reduced neuronal cell death [73]. Therefore, understanding the temporal and spatial dynamics of ERK1/2 signaling is crucial for exploiting its neuroprotective potential.

Besides ERK Signaling, p38 MAPK and JNK signaling pathways are also implicated in PD. Multiple experimental models have shown that exposure to α-synuclein aggregates or neurotoxins such as MPTP strongly activates p38 signaling in microglia, resulting in increased production of pro-inflammatory factors and reactive oxygen species [74,75,76]. Astrocytes similarly exhibit p38-dependent activation, leading to cytokine and reactive oxygen species release, thereby further expanding the contribution of glial cells to PD pathogenesis [77,78]. Notably, the pathogenic involvement of p38 MAPK extends beyond glial populations. Within dopaminergic neurons, p38 activation directly promotes neuronal death by impairing mitochondrial function, increasing oxidative stress, and triggering apoptotic signaling pathways [79]. Consistent with these observations, elevated p38 phosphorylation has been identified in substantia nigra neurons from patients with PD [79], indicating that both cell-intrinsic and extrinsic mechanisms converge on p38 signaling to drive neurodegeneration. For the role of JNK, postmortem analysis of PD brains has demonstrated increased phosphorylation of JNK within substantia nigra neurons [80]. This observation is supported by experimental studies showing that exposure to neurotoxins such as MPTP or 6-OHDA triggers robust and prolonged JNK activation that closely parallels the loss of dopaminergic neurons [81,82]. Notably, treatment with the JNK inhibitor SP600125 reduced dopaminergic neuronal apoptosis and preserved motor performance in MPTP-treated mice, underscoring the pivotal contribution of JNK signaling to PD pathogenesis [83]. Thus, p38 and JNK signaling constitute a pathogenic axis in PD that links chronic cellular stress to neuroinflammation and apoptotic neurodegeneration.

Collectively, the BDNF-ERK1/2 signaling axis is a central mediator of neuronal survival, synaptic integrity, and adaptive plasticity in the nigrostriatal system. Its impairment in PD contributes significantly to dopaminergic neurodegeneration. Re-establishing this pathway, either by augmenting BDNF availability or selectively targeting ERK1/2 signaling, holds great promise for neuroprotective therapy. Future studies should focus on elucidating the precise regulatory mechanisms governing ERK1/2 activation in dopaminergic neurons and developing targeted delivery systems capable of restoring trophic signaling in the diseased brain. A better understanding of the temporal dynamics and context-specific roles of ERK1/2 signaling will be essential for translating these insights into effective, disease-modifying treatments for PD.

## 10. Role of MAPK Signaling in Huntington’s Disease

HD represents one of the most devastating hereditary neurodegenerative diseases, resulting from a CAG repeat expansion in the huntingtin (HTT) gene that produces a mutant huntingtin protein (mHTT) with an elongated polyglutamine tract [84]. The disease is marked by selective degeneration of medium spiny neurons (MSNs) in the striatum, leading to characteristic motor impairments such as chorea and dystonia, accompanied by cognitive and psychiatric disturbances [84]. Despite decades of investigation, the precise molecular mechanisms connecting mHTT expression to neuronal death remain incompletely understood. Among various cellular pathways affected, neurotrophic signaling mediated by BDNF and its downstream effector ERK1/2 has received considerable attention due to its essential role in neuronal survival and plasticity.

BDNF is synthesized predominantly in cortical neurons and transported along corticostriatal projections to reach the striatum, where it supports the viability and function of MSNs [85]. Mutant huntingtin disrupts this process at multiple levels. Transcriptionally, mHTT sequesters the repressor-element 1-silencing transcription/neuron-restrictive silencer factor (REST/NRSF), a transcriptional repressor, inappropriately within the nucleus, repressing BDNF promoter activity [86]. Consequently, cortical neurons in HD exhibit markedly reduced BDNF mRNA and protein levels, which directly correlate with disease progression and striatal degeneration severity [86].

Axonal transport of BDNF-containing vesicles is similarly compromised. Wild-type huntingtin interacts with huntingtin-associated protein 1 (HAP1) and the dynein motor complex to facilitate efficient microtubule-based transport [86]. The polyglutamine-expanded form of huntingtin, however, has been shown to influence its capacity to interact with motor complex subunits and to transport intracellular cargoes, such as BDNF [87]. The resultant depletion of BDNF in the striatum deprives MSNs of essential trophic support, thereby reducing TrkB receptor activation and subsequent ERK1/2 phosphorylation. This early disruption in neurotrophic delivery constitutes one of the earliest detectable molecular events preceding overt neurodegeneration in HD.

Beyond the suppression of BDNF expression and availability by mHtt, TrkB receptor signaling itself is also disrupted by mHtt. BDNF-induced activation of the MEK/ERK pathway was found to be significantly diminished in striatal STHdhQ111 cells, while the activation of other TrkB downstream signaling cascades, such as Akt and PLCγ, remained unaffected [88]. Furthermore, the study demonstrated that attenuated BDNF- ERK1/2 pathway enhances the susceptibility of the mutant huntingtin striatal cells to oxidative stress, and that pharmacological stimulation of the MAPK pathway using phorbol 12-myristate 13-acetate (PMA) effectively protects against oxidative stress–induced cell death, underscoring the critical role of ERK1/2 signaling in mediating BDNF-dependent neuroprotection in STHdhQ111 striatal cells [88].

Postmortem analyses of HD brains, along with studies in transgenic mouse models, have demonstrated diminished ERK1/2-dependent gene expression, characterized by reduced pCREB and lower expression of pro-survival genes [89,90,91]. These findings emphasize the progressive loss of neuroprotective ERK1/2 signaling during disease development.

Given the critical role of the BDNF–ERK signaling pathway in maintaining neuronal viability and synaptic integrity, strategies aimed at restoring or augmenting this pathway have emerged as promising therapeutic directions for HD. The progressive impairment of BDNF production, TrkB receptor activation, and ERK1/2-dependent transcriptional regulation creates a cascade of neurodegenerative processes that can potentially be attenuated or reversed through molecular interventions. Several therapeutic approaches—ranging from neurotrophin supplementation to small-molecule enhancement of ERK1/2 signaling—have been explored in preclinical models of HD.

One of the most direct strategies for rescuing ERK1/2 signaling in HD involves restoring BDNF levels in the corticostriatal circuit. Viral vector-mediated delivery of BDNF has been among the most extensively studied approaches. For example, adeno-associated virus (AAV) to express BDNF in striatal neurons has demonstrated substantial neuroprotective effects in a transgenic HD rat model [92].

Another strategy involves pharmacological modulation of TrkB receptors to activate the downstream ERK1/2 cascade. Small-molecule TrkB agonists such as 7,8-dihydroxyflavone (7,8-DHF) have received considerable attention for their neuroprotective potential. 7,8-DHF crosses the blood–brain barrier efficiently and mimics BDNF by binding to the extracellular domain of TrkB, thereby triggering receptor dimerization and phosphorylation. In HD models, administration of 7,8-DHF stimulates both the PI3K/Akt and MAPK signaling pathways, promoting neuronal survival and plasticity while improving synaptic function [93,94]. Another promising small-molecule modulator of the ERK1/2 pathway is fisetin, a naturally occurring plant polyphenol. Fisetin was shown to increase ERK phosphorylation and enhance cell survival in PC12 cells expressing mHTT, while concurrently suppressing JNK and caspase-3 activation. Pharmacological inhibition of MEK abrogated both ERK1/2 activation and the neuroprotective effect of fisetin against mHTT-induced cell death, highlighting the pivotal role of ERK1/2 pathway activation in fisetin-mediated cellular rescue [95].

On the other hand, it is worth noting that several studies demonstrated the ERK signaling pathway was upregulated in multiple models of HD [89]. A study reported that the striatum of symptomatic 12-week-old transgenic R6/2 mice exhibited a marked increase in the number of pERK–immunoreactive cells relative to wild-type controls, accompanied by elevated levels of phosphorylated ERK-responsive transcription factors, including Elk-1 and CREB [96]. Expression of mutant Htt also results in sustained ERK activation in the rat pheochromocytoma PC12 cell line Htt14A2.5, with comparable effects observed in immortalized striatal ST14A cells [97]. Neuronal expression of Htt likewise elevates pERK levels in transgenic Drosophila. In contrast, in glial cells of transgenic Drosophila, expression of mutant Htt fragments or expanded polyQ proteins suppresses ERK phosphorylation, suggesting that in this cell type, polyQ peptides interfere with signaling events upstream of ERK [95]. These findings highlight that ERK signaling responses to Htt challenge are strongly context-dependent in HD pathogenesis.

Besides ERK signaling, p38 MAPK and JNK signaling pathways are also implicated in HD. In the R6/2 transgenic mouse model, enhanced p38 phosphorylation was detected in striatal neurons at symptomatic stages and was associated with mitochondrial impairment [98]. Likewise, YAC128 mice expressing full-length human mutant HTT exhibited increased p38 activity in striatal tissue, reinforcing the notion of a pathogenic role for this kinase [99]. Cell-based studies have further elucidated the contribution of p38 to HD pathology. In cultured striatal neurons from HD mice, NMDA receptor stimulation elicited excessive p38 activation that hastened apoptotic cell death [100]. Moreover, in human iPSC-derived neural cells harboring expanded CAG repeats, the neuronal death induced by BDNF withdrawal was prevented by SB23906, a selective inhibitor of activated p38, providing direct evidence that p38 signaling drives neuronal vulnerability in HD [101]. The involvement of the JNK pathway in HD pathology is also supported by multiple experimental studies. In a rat model, increased phosphorylation of the JNK substrate c-Jun was evident as early as 4 weeks after lentiviral expression of HTT171-82Q and remained elevated for up to 13 weeks [102]. In the same study, the use of several JNK inhibitory strategies—including a dominant-negative mutant of the upstream kinase MEKK1 (MEKK1^D1369A^) and the active JNK-binding domain of the scaffold protein JIP-1/IBI (IBI-JBD)—demonstrated that overexpression of MEKK1^D1369A^ or JIP-1/IBI mitigated polyQ-induced loss of the neuronal marker DARPP-32 [102]. Consistent with these observations, increased JNK pathway activation and c-Jun phosphorylation were also reported in R6/1 transgenic mice [103]. Cellular models further corroborate these findings. In immortalized rat hippocampal neurons, expression of full-length mutant HTT elicited pronounced JNK activation and accelerated apoptotic cell death [104]. Moreover, pharmacological inhibition of JNK with agents such as SP600125 suppressed caspase-3 activation and enhanced neuronal survival in PC12 and striatal cell models [97]. Thus, these data identify p38 and JNK as key mediators of neuronal degeneration in HD and underscore their potential as therapeutic targets.

Taken together, therapeutic strategies targeting the BDNF–ERK pathway offer a biologically grounded and mechanistically rational approach to counteract neurodegeneration in HD. Whether through exogenous BDNF delivery, pharmacological activation of TrkB, fine-tuned modulation of ERK1/2 activity, or epigenetic reactivation of neurotrophic genes, the restoration of this signaling axis holds promise for re-establishing neuronal resilience and delaying disease progression. The success of future interventions will depend on achieving precise control over the magnitude and timing of ERK1/2 activation—transforming this fundamental survival pathway into a clinically effective target for HD therapy.

## 11. Conclusions

Although AD, PD, and HD are traditionally discussed as distinct clinical and pathological entities, they share a common vulnerability in the BDNF–ERK signaling axis. BDNF–ERK signaling plays a central role in maintaining synaptic integrity, neuronal survival, and adaptive stress responses, rendering this pathway particularly sensitive to early synaptic dysfunction and oxidative stress—pathological features that are common to all three disorders. At the same time, the mechanisms that compromise BDNF–ERK signaling differ among diseases. In AD, amyloid-β and tau pathology preferentially disrupt synaptic BDNF signaling and downstream ERK activation, contributing to early synaptic loss. In PD, dopaminergic neurons experience heightened oxidative, mitochondrial stress, and α-synuclein pathology, which alters ERK signaling dynamics and interferes with BDNF-mediated neuroprotection. In HD, mHTT impairs both the transcription and axonal transport of BDNF, leading to insufficient activation of ERK-dependent survival pathways in vulnerable neuronal populations. Thus, BDNF–ERK signaling represents a shared neuroprotective pathway that is repeatedly challenged by overlapping pathological stresses but disrupted through disease-specific mechanisms. This integrated perspective reconciles similarities and differences among AD, PD, and HD and underscores the importance of preserving BDNF–ERK signaling integrity as a unifying therapeutic strategy across neurodegenerative diseases.

Across AD, PD, and HD, a unifying principle emerges in which the biological outcome of ERK1/2 signaling is critically determined by its temporal dynamics [70]. Transient and tightly regulated ERK1/2 activation, particularly when driven by physiological neurotrophic inputs such as the BDNF/TrkB pathway, is essential for neuronal maintenance, synaptic stability, and adaptive stress responses. In contrast, chronic or sustained ERK1/2 activation—frequently induced by pathological stimuli including protein aggregation, oxidative stress, mitochondrial dysfunction, and neuroinflammation—tends to promote maladaptive signaling cascades that exacerbate neuronal vulnerability and degeneration. In this review, transient ERK1/2 activation refers to a rapid and self-limiting phosphorylation event that peaks within minutes and returns to baseline within approximately one hour, typically downstream of physiological neurotrophic or synaptic signaling. By contrast, sustained ERK1/2 activation denotes prolonged phosphorylation persisting for several hours or longer, often extending to chronic activation observed under pathological conditions.

Beyond temporal dynamics, the divergent consequences of ERK1/2 activation can be further explained by differences in the subcellular localization of activated ERK and the regulation of ERK-specific phosphatases [70]. The spatial distribution of phosphorylated ERK1/2, particularly its cytosolic versus nuclear localization, critically influences downstream signaling outputs. Transient ERK1/2 activation accompanied by controlled cytosolic localization and timely signal termination supports adaptive gene expression and neuronal survival. In contrast, sustained ERK1/2 activation associated with prolonged nuclear accumulation has been linked to maladaptive transcriptional programs that contribute to neurodegeneration. A key determinant of these processes is the expression and localization of MAPK phosphatases (MKPs), including dual-specificity phosphatases such as DUSP1 (MKP-1), DUSP4 (MKP-2), and DUSP6 (MKP-3) [105]. Whereas DUSP1 and DUSP4 predominantly regulate nuclear ERK1/2 signaling, DUSP6 primarily controls cytosolic ERK1/2 activity. Dysregulation or mislocalization of these phosphatases can prolong ERK1/2 phosphorylation, disrupt spatial signal confinement, and facilitate the emergence of chronic ERK1/2 activation, as originally proposed in the concept of chronic ERK signaling. Such alterations in phosphatase-mediated ERK regulation may therefore underlie why ERK activation appears neuroprotective in some experimental models yet detrimental in others.

Importantly, ERK1/2 activation in neurodegenerative disorders should not be viewed exclusively as either a cause or a consequence of disease progression. In early or preclinical stages, ERK1/2 signaling may primarily reflect a compensatory response aimed at preserving neuronal function in the face of accumulating pathological stress. However, as disease advances and ERK1/2 activation becomes prolonged and uncoupled from physiological neurotrophic regulation, this signaling pathway may transition into an active driver of pathology. Such chronic ERK1/2 activation can amplify oxidative stress, disrupt synaptic homeostasis, and interfere with neuronal survival programs, thereby accelerating disease progression across AD, PD, and HD. These considerations underscore the necessity of interpreting ERK1/2 signaling within a dynamic, stage-dependent framework. Accordingly, therapeutic strategies targeting the ERK pathway should aim to restore appropriate temporal and spatial control rather than indiscriminately enhancing or suppressing ERK1/2 activity.

**Figure 2:**
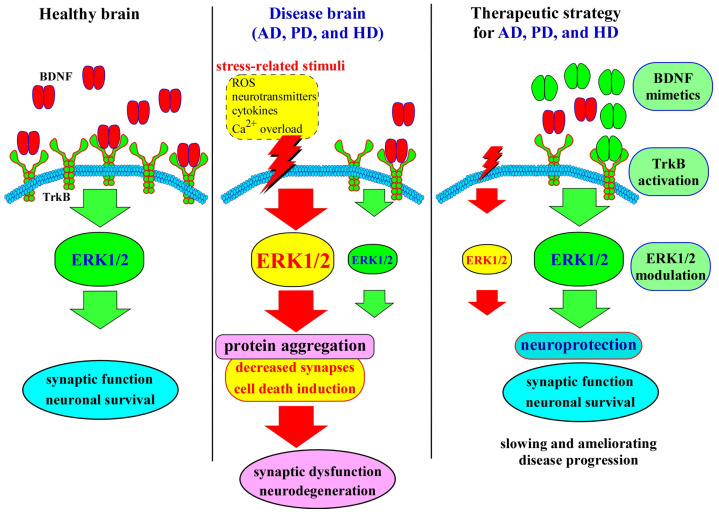
Summary of BDNF–ERK signaling and its relevance to major neurodegenerative diseases. The schematic illustrates how BDNF activates ERK signaling through TrkB receptor engagement, supporting neuronal survival and synaptic plasticity (left panel). Dysregulation of this pathway contributes to pathological outcomes across neurodegenerative disorders (middle panel). In AD, reduced BDNF–ERK signaling is associated with synaptic loss and cognitive decline. In PD, impaired pathway activity increases dopaminergic neuronal vulnerability within the nigrostriatal system. In HD, deficits in BDNF transport and ERK activation promote striatal neurodegeneration. The right panel summarizes key therapeutic strategies aimed at restoring pathway function, including enhancing BDNF expression, activating TrkB receptors, and modulating ERK activity.

Therapeutically, restoring or enhancing BDNF–ERK signaling represents a compelling strategy. Preclinical evidence suggests that interventions capable of increasing BDNF availability, activating TrkB receptors, or directly modulating ERK1/2 activity can ameliorate synaptic deficits, reduce neuronal loss, and improve functional outcomes (Figure 2). Nonetheless, translating these findings into clinical therapies remains challenging. Achieving spatiotemporally precise activation of ERK1/2 without eliciting off-target effects, maladaptive plasticity, or oncogenic risks requires sophisticated delivery systems and a nuanced understanding of the signaling dynamics in different neuronal populations.

Especially, any therapeutic manipulation of ERK1/2 signaling must carefully consider its well-established oncogenic potential. Sustained, growth factor–independent ERK1/2 activation is a hallmark of many cancers, underscoring the risks associated with indiscriminate ERK activation [106]. Therefore, ERK1/2 modulation cannot be regarded as universally therapeutic. Instead, the therapeutic window likely resides in restoring physiological, transient, and spatially confined ERK1/2 signaling downstream of neurotrophic pathways such as BDNF/TrkB, rather than promoting chronic pathway activation. This distinction highlights that the goal of ERK-targeted interventions in neurodegenerative diseases should be signal normalization rather than pathway amplification, thereby minimizing oncogenic risk while preserving neuroprotective functions.

Future research should focus on several key directions. First, it is critical to delineate disease-specific mechanisms of BDNF–ERK dysregulation, including the impact of protein aggregates, genetic mutations, and environmental stressors on pathway integrity. Second, integrating multi-omics approaches—encompassing transcriptomics, proteomics, and phosphoproteomics—may reveal novel regulatory nodes and biomarkers predictive of pathway dysfunction and therapeutic responsiveness. Third, combinatorial strategies that simultaneously target BDNF–ERK signaling and intersecting pathways, such as PI3K/Akt, JNK, and p38MAPK, may offer synergistic neuroprotection, particularly in multifactorial conditions like AD and PD. Additionally, emerging technologies, including gene therapy, engineered neurotrophic mimetics, and nanocarrier-based delivery, hold promise for achieving sustained, targeted modulation of this pathway in specific neuronal populations.

Finally, longitudinal and translational studies are needed to evaluate the efficacy and safety of BDNF–ERK-targeted interventions in human patients. Understanding the temporal window during which pathway restoration is most effective, as well as the long-term effects on synaptic function and cognitive or motor outcomes, will be essential for clinical application. By integrating mechanistic insights with advanced therapeutic approaches, targeting the BDNF–ERK axis offers a realistic avenue not only for slowing neurodegeneration but also for potentially restoring neuronal function and improving quality of life in patients with Alzheimer’s, Parkinson’s, and Huntington’s disease.

## Data Availability

No new data were created or reported in this review article.
